# Altered resting-state brain activity in Parkinson’s disease patients with freezing of gait

**DOI:** 10.1038/s41598-017-16922-0

**Published:** 2017-12-01

**Authors:** Tao-Mian Mi, Shan-Shan Mei, Pei-Peng Liang, Lin-Lin Gao, Kun-Cheng Li, Tao Wu, Piu Chan

**Affiliations:** 1 Department of Neurology, Neurobiology and Geriatrics, Xuanwu Hospital of Capital Medical University, Beijing Institute of Brain Disorders, Beijing, 100053 China; 2 Department of Radiology, Xuanwu Hospital of Capital Medical University, Beijing, 100053 China; 3 Beijing Key Laboratory of MRI and Brain Informatics, Beijing, 100053 China; 4 Clinical Center for Parkinson’s Disease, Capital Medical University, Beijing, 100053 China; 5 Key Laboratory for Neurodegenerative Disease of the Ministry of Education, Beijing Key Laboratory for Parkinson’s Disease, Beijing, 100053 China; 6 National Clinical Research Center for Geriatric Disorders, Beijing, 100053 China

## Abstract

Freezing of gait (FOG) is a common and debilitating symptom in Parkinson’s disease (PD). The current study investigated alterations of resting-state spontaneous brain activity in PD patients with FOG. A total of 29 patients with FOG, 28 patients without FOG and 31 controls were included. All subjects underwent resting-state functional MRI, and the amplitude of low-frequency fluctuation (ALFF) was calculated to measure the spontaneous brain activity. Between-group differences and correlations with FOG severity (both subjective and objective measures) were analyzed. Compared to those without FOG, patients with FOG showed increased ALFF in right anterior cingulate cortex (ACC) and left inferior parietal lobule (IPL), as well as decreased ALFF in right superior frontal gyrus (SFG), bilateral cerebellum and left thalamus. Correlation analyses demonstrated that ALFF within the right SFG, right ACC and bilateral pallidum were positively correlated with FOG; while ALFF within the thalamus, putamen, cerebellum and sensorimotor regions were negatively correlated. Our results indicate that FOG is associated with dysfunction within frontal-parietal regions, along with increased inhibitory outputs from basal ganglia. Additionally, altered activity of cerebellum implicates its role in the pathophysiology of FOG. These findings provide further insight into the underlying neural mechanisms of FOG in PD patients.

## Introduction

Freezing of gait (FOG) is described as a ‘brief, episodic absence or marked reduction of forward progression of the feet despite the intention to walk’^1^, generally occurring during gait initiation and/or turning^2^. FOG is one of the most debilitating symptoms in Parkinson’s disease (PD), contributing to falls and reduced mobility and quality of life^2,3^. Common treatments such as anti-Parkinson medication do not consistently provide adequate benefit^4^. FOG is usually observed in the advanced stages of PD, but it can also present at early phase^2^. It has been suggested that FOG is not associated with the cardinal features of PD (tremor, bradykinesia or rigidity)^4,5^, but significantly associated with other factors, such as postural instability^6^ and impaired executive function^7^.

Although it is difficult to accompany gait into neuroimaging, much insight into FOG has been gained from neuroimaging techniques^8^. Using virtual reality and motor imagery paradigms, recent task-based functional MRI (fMRI) studies reported decreased neural activity within the bilateral sensorimotor regions and a concomitant increased response within fronto-parietal cortical regions in PD patients who have FOG compared to those without^9,10^. Decreased neural response had also been observed in a number of subcortical nuclei, including the bilateral caudate head, thalamus, subthalamic nucleus and globus pallidus internus during FOG episodes^9^. In addition, one previous multimodal study using resting-state fMRI and diffusion tensor imaging demonstrated altered functional and structural connectivity in the mesencephalic locomotor region and cerebellar locomotor region, involving mainly those connecting the peduncolopontine nucleus with the frontal cortices and cerebellum^11,12^.

While the above-mentioned studies provided essential information to unravel the neural correlates of FOG in PD, current knowledge of the underpinnings underlying FOG remains limited. It has been suggested that cerebral alternations observed in the resting-state, in the absence of experimental tasks, can take full advantage of the neural origin of spontaneous blood-oxygen-level-dependent (BOLD) signal fluctuations^13^. To date, several resting-state fMRI studies have investigated FOG related functional connectivity changes in PD patients, showing altered functional connectivities not only in the locomotor network^11^, but also in “cognitive” circuits such as the executive-attention related frontoparietal and visual occipito-temporal networks^14^. Although the result of abnormal functional connectivity between two remote areas is comprehensive and integrative, one could not draw any conclusion about which area is abnormal from such an examination^15^. Regarding the amplitude of low-frequency fluctuation (ALFF), Zang *et al*.^15^ proposed that ALFF measures the amplitude of low-frequency (0.01~0.08 Hz) BOLD signal and can be used as an index of local spontaneous neural activity in resting state. In addition, this technique provides insight into the neural substrates underlying PD at a local level^15,16^, which may have further implications for identifying treatment targets and thus guiding the development of new therapies for brain stimulation.

To our knowledge, no studies have investigated the spontaneous brain activity related to FOG in PD patients in the resting-state. ALFF has been used to study neurophysiological mechanisms in PD patients with depression^17^, mild cognitive impairment^16^ and rapid eye movement sleep behavior disorder^18^. Accordingly, we used ALFF to study the spontaneous brain activity in PD patients with FOG. Furthermore, we also explored the neural correlates with several gait deficits which are more impaired or specific in patients with FOG when comparing to patients without FOG and healthy controls in the gait analysis.

## Results

### Clinical and demographic characteristics

Participant demographics and clinical features are described in Table 1. Briefly, patients with FOG had longer disease duration, more severe parkinsonism as assessed by the Hoehn and Yahr (H-Y) stage and Movement Disorder Society Unified Parkinson’s Disease Rating Scale motor scores (MDS-UPDRS III), and higher levodopa equivalent daily dose (LEDD). Therefore, we controlled for these factors in the further analysis. There were no significant differences in onset age and onset side between patients with and without FOG. In the FOG group, the mean FOG duration was 1.95 ± 1.62 years, while the mean new freezing of gait questionnaire (NFOGQ) Part II and Part III scores were 15.13 ± 3.32 and 5.77 ± 2.32 respectively. Both patients with and without FOG had higher Hamilton Depression Rating Scale (HAMD) scores than healthy controls, whereas no difference was found between the two disease groups. Other clinical variables were similar among the three groups, including sex, age, Mini-Mental State Examination (MMSE), Montreal Cognitive Assessment (MoCA) total score and Executive Function sub-score.

**Table 1:** Demographic and clinical features of all participants.

Features	Patients with FOG (n = 31)	Patients without FOG (n = 31)	Healthy controls (n = 32)	F/T	p-value
Sex (m/f)	14/17	20/11	17/15	2.365	0.307^a^
Age, years	60.87 ± 8.14	58.03 ± 9.78	58.34 ± 7.25	1.057	0.352^b^
Disease Duration, years	7.90 ± 5.06	5.23 ± 3.46	—	2.430	0.018^c*^
Onset Age, years	52.68 ± 8.23	52.84 ± 10.56	—	−0.067	0.947^c^
Onset Side (R/L/B)	11/15/5	10/18/3	—	0.820	0.664^a^
H-Y stage (OFF)	2.56 ± 0.73	1.92 ± 0.62	—	3.757	0.000^c*^
MDS-UPDRS III (OFF)	41.48 ± 17.18	30.81 ± 14.41	—	2.651	0.010^c*^
FOG duration, years	1.95 ± 1.62	—			
NFOGQ-II	15.13 ± 3.32	—	—	—	—
NFOGQ-III	5.77 ± 2.32	—	—	—	—
LEDD, mg/d	704.67 ± 381.15	396.67 ± 296.06	—	3.495	0.000^c*^
MMSE	28.48 ± 2.03	27.84 ± 1.73	28.59 ± 1.43	1.704	0.188^b^
MoCA	25.58 ± 3.66	24.81 ± 2.99	26.16 ± 3.41	1.274	0.285^b^
Executive Function sub-score	3.23 ± 0.99	3.03 ± 0.96	3.13 ± 0.98	0.296	0.744^b^
HAMD	7.74 ± 4.70	6.32 ± 4.90	3.03 ± 2.92	10.180	0.000^b*^

Means and SD are shown for continuous variables. FOG: freezing of gait; Onset Side (R/L/B): Right/Left/Bilateral onset; H-Y stage: Hoehn and Yahr stage; MDS-UPDRS III: Movement Disorder Society Unified Parkinson’s Disease Rating Scale motor score; NFOGQ: new freezing of gait questionnaire; LEDD: levodopa equivalent daily dose; MMSE: Mini-Mental State Examination; MoCA: Montreal Cognitive Assessment; HAMD: Hamilton Depression Scale. ^a^chi-square test; ^b^variance analysis; ^c^two independent sample t-test.

### Gait performances

Descriptive statistics of the gait performances during the Instrumented Stand and Walk test are shown in Table 2. Briefly, both patients with and without FOG had significantly reduced first step range of motion, stride length, stride velocity and turn peak velocity compared to healthy control subjects. Furthermore, *post-hoc* t-tests demonstrated that all of these measures were more significantly impaired in patients with FOG than in those without FOG. *Post-hoc* t-tests also revealed that patients with FOG had significantly longer total duration and turn duration, more turn steps, as well as more reduced arm swing amplitude compared to both healthy controls and patients without FOG. We report no differences in the other parameters among the three groups.

**Table 2:** Descriptive statistics of gait performance in all participates.

Measurements	Patients with FOG	Patients without FOG	Healthy controls	F	p	p1	p2	p3
**Total Duration**	59.01 ± 25.87	46.99 ± 6.33	42.07 ± 1.80	10.130	0.000*	0.003^#^	0.000^#^	0.206
**Step Initiation**								
APA duration (s)	0.51 ± 0.24	0.58 ± 0.32	0.56 ± 0.31	0.598	0.552	0.292	0.445	0.764
APA latency (s)	0.83 ± 1.27	0.68 ± 0.95	0.36 ± 0.16	2.148	0.123	0.515	0.045	0.174
First step range of motion (degrees)	22.80 ± 9.27	32.94 ± 9.63	40.61 ± 7.42	32.289	0.000*	0.000^#^	0.000^#^	0.001^#^
First step latency (s)	0.83 ± 1.27	0.68 ± 0.95	0.36 ± 0.16	2.148	0.123	0.515	0.045	0.174
**Straight Walking**								
Stride length (m)	0.98 ± 0.29	1.26 ± 0.18	1.40 ± 0.09	34.927	0.000*	0.000^#^	0.000^#^	0.006^#^
Stride velocity (m/s)	0.94 ± 0.27	1.16 ± 0.19	1.34 ± 0.09	33.357	0.000*	0.000^#^	0.000^#^	0.000^#^
Cadence (steps/min)	118.92 ± 22.00	110.53 ± 8.71	115.00 ± 8.14	2.640	0.077	0.024^#^	0.282	0.221
Double support time (% gait cycle)	23.65 ± 8.02	21.71 ± 4.66	20.91 ± 4.08	1.831	0.166	0.192	0.066	0.590
Swing time (% gait cycle)	38.17 ± 4.01	39.15 ± 2.33	39.54 ± 2.04	1.831	0.166	0.192	0.066	0.590
Stance time (% gait cycle)	61.83 ± 4.01	60.85 ± 2.33	60.46 ± 2.04	1.831	0.166	0.192	0.066	0.590
Arm swing amplitude (degrees)	9.73 ± 6.65	14.34 ± 9.40	16.79 ± 10.56	4.907	0.010*	0.049^#^	0.003^#^	0.290
**Turning**								
Turn duration (s)	5.34 ± 3.21	3.03 ± 0.71	2.28 ± 0.50	21.710	0.000*	0.000^#^	0.000^#^	0.123
Turn steps	9.89 ± 4.09	6.00 ± 1.28	4.88 ± 0.93	34.060	0.000*	0.000^#^	0.000^#^	0.080
Turn peak velocity (degrees/sec)	100.46 ± 39.39	129.07 ± 24.57	164.79 ± 36.51	28.133	0.000*	0.001*	0.000*	0.000*

FOG: freezing of gait; APA: anticipatory postural adjustments. p1: patients with FOG vs patients without FOG; p2: patients with FOG vs healthy controls; p3: patients without FOG vs healthy controls. *p < 0.001 in the ANOVA analyses; ^#^p < 0.05 in the *post-hoc* analyses.

### Comparison of ALFF values among groups

As mentioned in the Method, we excluded 6 subjects (2 patients with FOG, 3 patients without FOG and 1 healthy control) due to the remarkable head motion. Thus, our final ALFF analysis included 29 patients with FOG, 28 patients without FOG, and 31 healthy controls.

**Figure 1:**
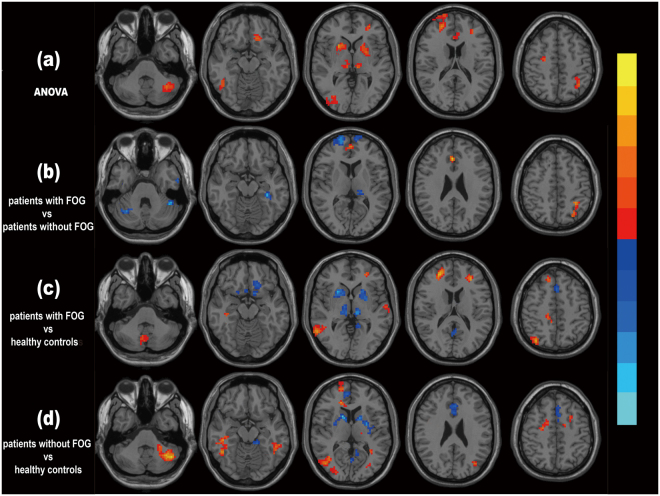
Differences in ALFF values among groups. **(a)** ANOVA results of the ALFF values among the three groups**. (b)** *Post-hoc* analysis showing differences in ALFF values between patients with FOG and without FOG, with MDS-UPDRS III score, H-Y stage, disease duration and LEDD as covariates. **(c)** *Post-hoc* analysis showing differences in ALFF values between patients with FOG and healthy controls. **(d)** *Post-hoc* analysis showing differences in ALFF values between patients without FOG and healthy controls. Brain regions presenting with significantly increased ALFF values are shown in hot colors, whereas decreased ALFF values are shown in cold colors. P value thresholds were set at a corrected P < 0.05 with a voxel-level p < 0.001, determined by AlphaSim correction.

Figure 1a shows the brain regions presenting with significant differences in the ANOVA analysis. The three groups had significantly different ALFF values in the following regions: bilateral putamen and thalamus, left anterior cingulate cortex, Crus I of the left cerebellum, right superior frontal gyrus (SFG), left SFG (orbital part), left middle frontal gyrus, right inferior temporal gyrus, left inferior parietal lobule (IPL), right precentral gyrus and right middle occipital gyrus.

Figure 1b–d shows the brain regions presenting with significant differences in the *post-hoc* analysis. Compared to those without FOG, patients with FOG had decreased ALFF values in the bilateral Crus I of cerebellum, bilateral SFG and left thalamus; along with increased ALFF values in the right anterior cingulate cortex and left IPL. Comparison between patients with FOG and healthy controls revealed that, ALFF values in the bilateral putamen and thalamus, left inferior frontal gyrus (orbital part), left supplementary motor area and left precuneus were decreased; while ALFF values in the lobule VIII of vermis, left middle frontal gyrus, right SFG, right superior parietal lobule and right middle temporal gyrus were increased. *Post-hoc* t test also demonstrated that relative to healthy controls, patients without FOG had decreased ALFF values in the bilateral putamen and anterior cingulate cortex, left supplementary motor area and right precuneus; and increased ALFF values in the left Crus I of cerebellum, right SFG, right middle occipital gyrus and bilateral inferior temporal gyrus (Table 3).

**Table 3:** Differences of ALFF values among the groups.

Brain regions (AAL atlas)	MNI coordinates			T value	Cluster size (mm^3^)
	X	Y	Z		
**ANOVA result**					
Thalamus_R	13	−22	1	4.401	137
Occipital_Mid_R	30	−84	2	4.718	65
Cingulum_Ant_L	−3	21	30	5.163	107
Temporal_Inf_R	45	−55	−14	5.677	78
Precentral_R	30	−9	54	6.274	118
Parietal_Inf_L	−36	−54	51	6.947	45
Putamen_L	−20	3	10	7.079	260
Frontal_Mid_L	−30	48	3	7.321	65
Cerebellum_Crus1_L	−36	−75	−30	7.437	210
Thalamus_L	−18	−21	15	8.368	53
Frontal_Sup_R	24	48	18	8.939	149
Frontal_Sup_Orb_L	−15	27	−18	9.575	55
Putamen_R	24	15	−3	13.457	494
**Patients with FOG vs patients without FOG**					
Thalamus_L	−9	−18	15	−2.930	39
Cerebellum_Crus1_L	−44	−47	−30	−3.293	284
Frontal_Sup_R	30	63	15	−3.604	113
Cerebellum_Crus1_R	36	−63	−27	−4.140	53
Parietal_Inf_L	−33	−48	51	3.257	55
Cingulum_Ant_R	6	42	3	3.584	165
**Patients with FOG vs healthy controls**					
Precuneus_L	0	−54	21	−2.893	66
Supp_Motor_Area_L	0	21	48	−3.228	38
Putamen_L	−23	6	−6	−3.316	239
Thalamus_R	12	−14	8	−3.432	126
Frontal_Inf_Orb_L	−18	27	−18	−3.840	122
Thalamus_L	−10	−20	8	−3.936	167
Putamen_R	24	15	−3	−4.626	370
Vermis_8	5	−64	−36	2.612	48
Temporal_Mid_R	60	−54	0	3.575	108
Parietal_Sup_R	36	−72	51	3.814	49
Frontal_Mid_L	−30	39	15	3.968	137
Frontal_Sup_R	24	48	18	4.064	90
**Patients without FOG vs healthy controls**					
Putamen_R	23	8	−1	−2.710	66
Cingulum_Ant_R	4	21	28	−2.799	96
Cingulum_Ant_L	−3	20	26	−2.824	125
Putamen_L	−21	8	0	−2.952	106
Supp_Motor_Area_L	−3	−3	63	−3.359	57
Precuneus_R	0	−48	72	−4.135	160
Temporal_Inf_L	−48	−43	−15	2.952	138
Temporal_Inf_R	49	−51	−15	3.120	124
Occipital_Mid_R	34	−84	3	3.895	563
Frontal_Sup_R	24	48	18	3.961	801
Cerebellum_Crus1_L	−33	−75	−30	4.147	424

MNI: Montreal Neurological Institute; AAL: Automated Anatomical Labeling.

### Relationships between ALFF values and FOG severity in patients with FOG

**Figure 2:**
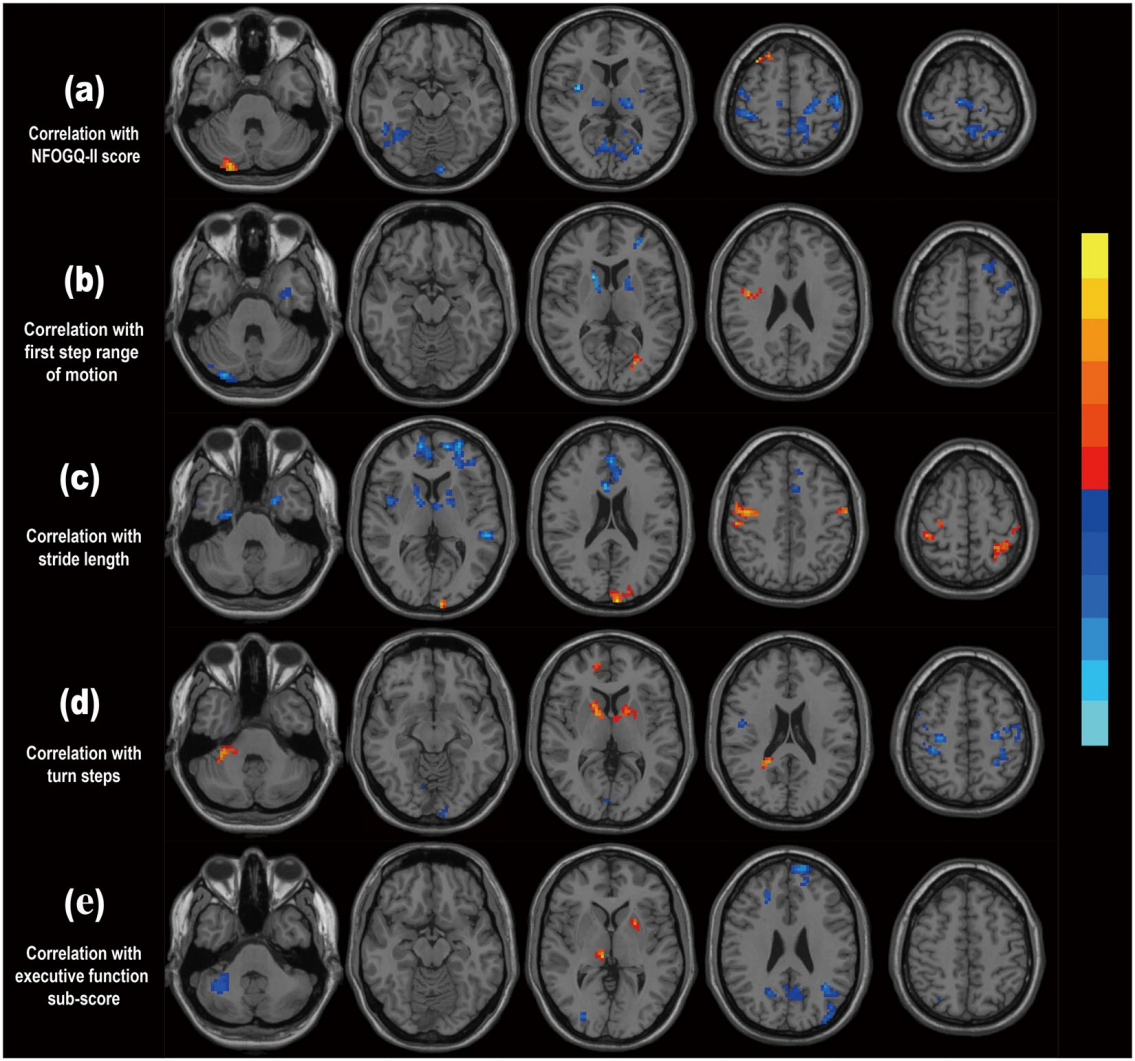
Correlation between ALFF values and FOG severity as well as Executive Function sub-score in PD patients with FOG. **(a)** Pearson’s correlation analysis between ALFF values and NFOGQ-II score. **(b)** Pearson’s correlation analysis between ALFF values and first step range of motion; **(c)** Pearson’s correlation analysis between ALFF values and stride length; **(d)** Pearson’s correlation analysis between ALFF values and turn steps; **(e)** Pearson’s correlation analysis between ALFF values and Executive Function sub-score. Brain regions showing a significantly increased positive correlation are shown in hot colors, whereas those showing a negative correlation are shown in cold colors. P value thresholds were set at a corrected P < 0.05 with a voxel-level p < 0.001, determined by AlphaSim correction.

Figure 2a–d shows brain regions that were proven to be significantly correlated with four measures for FOG severity in the FOG group, including one subjective measure (NFOGQ-II) and three relative objective measures/gait parameters (first step range of motion, stride length and turn steps).

Correlation analysis between ALFF values and NFOGQ-II showed that ALFF values in the bilateral putamen, thalamus and precentral gyrus, right supplementary motor area, left precuneus, right inferior temporal gyrus and left IPL were negatively correlated with NFOGQ-II score; whereas ALFF values in the right Crus II of cerebellum, left anterior cingulate cortex and right SFG were positively correlated with NFOGQ-II score. Correlation analysis between ALFF values and first step range of motion demonstrated a negative correlation in bilateral globus pallidus, left SFG and left precentral gyrus. Correlation analysis between ALFF values and stride length showed a negative correlation in bilateral SFG and globus pallius, left anterior cingulate cortex, right insula and left middle temporal gyrus; and a positive correlation in bilateral postcentral gyrus, left superior parietal cortex and left superior occipital gyrus. Correlation between ALFF values and turn steps revealed a negative correlation in bilateral postcentral gyrus, left inferior parietal cortex and left precuneus, as well as a positive correlation in bilateral globus pallidus and right SFG (Table 4).

**Table 4:** Correlation between ALFF values and FOG severity in PD patients with FOG.

Brain regions (AAL atlas)	MNI coordinates			T value	Cluster size (mm^3^)
	X	Y	Z		
**Correlation between ALFF values and NFOGQ-II score**					
Parietal_Inf_L	−45	−39	51	−0.523	83
Supp_Motor_Area_R	7	−19	67	−0.530	115
Precuneus_L	−9	−46	60	−0.548	130
Putamen_L	−30	−6	6	−0.566	36
Thalamus_R	9	−21	6	−0.587	46
Thalamus_L	−15	−18	12	−0.589	44
Temporal_Inf_R	39	−60	−6	−0.624	168
Precentral_R	48	−9	48	−0.641	424
Putamen_R	33	−3	9	−0.723	75
Precentral_L	−45	−6	30	−0.723	303
Cingulum_Ant_L	−9	39	0	0.502	36
Cerebellum_Crus2_R	24	−90	−27	0.581	50
Frontal_Sup_R	30	33	54	0.616	86
**Correlation between ALFF values and first step range of motion**					
Pallidum_L	−19	7	6	−0.481	61
Precentral_L	−30	−3	46	−0.496	50
Precentral_L	−30	−3	46	−0.496	50
Frontal_Sup_L	−15	24	51	−0.583	42
Temporal_Mid_L	−54	−30	3	−0.641	59
Pallidum_R	18	12	9	−0.650	40
Cerebellum_Crus1_R	27	−88	−27	−0.696	97
**Correlation between ALFF values and stride length**					
Pallidum_L	−19	−1	5	−0.454	58
Pallidum_R	16	5	5	−0.473	65
Insula_R	43	0	5	−0.517	62
Frontal_Sup_R	10	62	8	−0.543	168
Cingulum_Ant_L	−4	42	21	−0.554	248
Temporal_Mid_L	−54	−33	6	−0.665	78
Frontal_Sup_L	−27	57	6	−0.672	331
Parietal_Sup_L	−28	−45	61	0.515	128
Occipital_Sup_L	−7	−99	21	0.581	195
Postcentral_L	−57	−9	39	0.604	135
Postcentral_R	42	−9	48	0.621	234
**Correlation between ALFF values and turn steps**					
Parietal_Inf_L	−27	−45	54	−0.509	52
Postcentral_L	−48	−9	40	−0.537	154
Precuneus_L	−6	−57	66	−0.599	54
Postcentral_R	48	−6	28	−0.666	172
Pallidum_L	−17	5	2	0.520	92
Frontal_Sup_R	21	21	63	0.587	48
Pallidum_R	13	4	2	0.692	108

MNI: Montreal Neurological Institute; AAL: Automated Anatomical Labeling.

### Relationships between ALFF values and Executive Function sub-score in patients with FOG

Figure 2e exhibits the brain regions that were significantly correlated with the Executive Function sub-score. Correlation analysis between ALFF values and Executive Function sub-score showed a negative correlation in the right Crus I of cerebellum, left SFG, left precuneus and left middle occipital gyrus, as well as a positive correlation in the left putamen and right thalamus (Table 5).

**Table 5:** Correlation between ALFF values and Executive Function sub−score in PD patients with FOG.

Brain regions (AAL atlas)	MNI coordinates			T value	Cluster size (mm^3^)
	X	Y	Z		
Precuneus_L	−5	−58	26	−0.416	111
Cerebellum_Crus1_R	36	−55	−38	−0.430	173
Occipital_Mid_L	−36	−88	26	−0.482	257
Frontal_Sup_L	−13	61	26	−0.485	96
Thalamus_R	11	−18	0	0.482	73
Putamen_L	−24	9	0	0.599	50

MNI: Montreal Neurological Institute; AAL: Automated Anatomical Labeling.

## Discussion

The current study is the first to investigate the pattern of resting-state spontaneous brain activity in PD patients with FOG. In the present study, we used inertial sensors to acquire gait parameters during gait which is powerful and also permits more precise and specific correlations between clinical data and functional MRI results. Results presented here demonstrate that FOG is associated with decreased ALFF value in the prefrontal cortex, as well as increased ALFF values in the parietal cortex and anterior cingulate cortex (ACC). In addition, we also found that FOG is positively correlated with ALFF values in the bilateral globus pallidus and negatively correlated with ALFF values in the bilateral sensorimotor regions and thalamus. These findings indicate that FOG in PD is likely to be due to the impaired processing in the frontal and parietal regions^8^, and may also be associated with increased basal ganglia inhibitory output, which lead to decreased information processing in the thalamus and brainstem^9,19^. Interestingly, we also found that altered activity of Crus I of cerebellum plays a critical role in the pathophysiology of FOG.

The key spatiotemporal findings observed during straight walking in our study are consistent with those from most previous studies, demonstrating that patients with FOG walk more slowly with a shorter stride length than those without FOG^20,21^. No significant difference in cadence was found among the groups, indicating that PD patients are capable of modulating cadence, thereby at least partly compensating for their smaller step length^22,23^. Reduced arm swing amplitude is reflective of bradykinesia in the upper body, and has been reported to be associated with an increased risk of falls for patients with PD^24^. Patients with FOG have significantly reduced arm swing amplitude, which may be associated with the more advanced disease stage^25^. It has been suggested that patients with FOG spend more time in the double-support phase of gait^26^. Although not significant, a tendency of longer double support time was documented in our study, which could be due to the fact that patients were only moderately affected by FOG. With respect to gait initiation, patients with FOG show a much lower first step range of motion compared to patients without FOG. Unlike the spatial control of APAs, relative timing (APAs duration and latency, first step latency) is unaffected in PD patients^27^. As expected, turning in patients with FOG is characterized by slow velocity, additional steps and time needed to complete the turn^28^. Our results provide further evidence for the pronounced impairments during gait initiation and turning in patients with FOG^2^.

In patients with FOG, there was a decreased ALLF value in the right SFG and a concomitant increased ALLF value in the left IPL compared to patients without FOG. Our findings are consistent with a number of previous neuroimaging experiments that have implicated frontoparietal dysfunction in the pathophysiology of freezing^8,9,14^. The prefrontal and posterior parietal cortices have previously been suggested to co-activate as a functional network, described as the cognitive control network^29^. One recent task-based fMRI study demonstrated that regions within this network are significantly activated during freezing episodes, which underlies a compensatory recruitment of regions that are attempting to overcome a freezing episode^9^. Although both of the two PD groups had higher activity in the right SFG than healthy controls, the less increased ALFF value in patients with FOG might represent an unsatisfied or failing compensatory mechanism. Moreover, there was a positive correlation between the ALFF value in the right SFG and the severity of the FOG. These findings indicate that the spontaneous neural activity in the right SFG is decreased in patients with FOG compared to those without, and this decreased activity becomes more significant as FOG progresses. On the other hand, theoretically, the increased ALFF in the left IPL in patients with FOG might reflect a compensatory or pathological effect when comparing to patients without FOG. However, pathological impairments should be more severe as FOG progresses, which is inconsistent with our finding of the negative correlations with the NFOGQ-II score and turn steps. Therefore, we suggested that the increased ALFF value in the left IPL is more likely a reflection of the compensatory effect; however, this effect becomes less significant as the symptom progresses. Of note, a previous resting-state fMRI study^14^ revealed that patients with FOG exhibit significantly reduced functional connectivity in the frontal and parietal regions. These findings indicate that not only the functional connectivity but also the spontaneous neural activity within the frontal and parietal regions is impaired in patients with FOG.

Our results also show that the ALFF value in the left thalamus was significantly decreased in patients with FOG when comparing to patients without FOG. In addition, the ALFF values in the bilateral thalamus and putamen were negatively correlated with FOG severity. The thalamus and brainstem locomotor region, particularly the pedunculopontine nucleus, are the major terminals of the corticostriatal projections, receiving inhibitory outputs arising from the basal ganglia. Lewis and Barker proposed an “interference model” and explained the occurrence of FOG as a momentary breakdown of concurrent information processing of those competing yet complementary tasks, such as cognitive and limbic load during motor tasks. Decreased neural reserve in the basal ganglia leads to a “cross-talk” within these competing inputs; consequently, a paroxysmal excessive inhibition of the thalamus and pedunculopontine nucleus are induced, thus triggering a freezing episode^19^. The negative correlation between ALFF value in the left thalamus and the NFOGQ-II score indicates that this dysfunction becomes more pronounced as the FOG progresses.

The cerebellum is one of the major subcortical structures that influence multiple aspects of motor, cognitive and affective behavior^30^. Growing evidence suggests that the cerebellum plays a major role in the pathophysiology of PD, including both pathological and compensatory effects^31^. However, the implication of cerebellar dysfunction in FOG is rare. One previous diffusion tensor imaging study demonstrated that FOG is associated with poor white matter connectivity between the pedunculopontine nucleus and the cerebellum^12^. A more recent study utilizing the lesion network mapping technique identified that lesions causing FOG are located within a common functional network characterized by connectivity to the cerebellar locomotor region^32^. Among the cerebellum’s complicated lobular division, Crus I and II of cerebellum sends and receives projections from prefrontal cortex area^33^, forming a closed-loop circuit and linking to association networks involved with executive control^34^. We found that patients with FOG, in the present study, had significantly lower ALFF value in the bilateral Crus I of cerebellum than patients without FOG, whereas had no significant difference with healthy controls. Previous studies showed that during the cognitive performance, metabolism in the cerebellum is increased in PD patients^35^, which might play a compensatory effort to maintain the cognitive function^12^. Due to the cognitive feature of FOG^36–38^, we therefore proposed that, presumably, the failure to increase activity for the Crus I of cerebellum might reflect its impaired compensatory effort in patients with FOG. Our findings provide evidence that cerebellar dysfunction might also play a role in the pathophysiology of FOG, however, further investigations are needed to clarify the role of cerebellum plays in FOG.

Functions of the ACC are central to intelligent behaviors, including emotional self-control, focused problem solving, adaptive response to changing conditions, and switching action plans^39–41^. It has been suggested that dysfunctions within this area might predispose individuals to FOG^42^. Our finding of increased ALFF value in the right ACC in patients with FOG compared to those without FOG is consistent with the results from a previous task-based fMRI study^9^. This increased ALFF might be a compensatory effect to improve the limited ability to switch between motor programs in patients with FOG. The positive correlation of ALFF with the NFOGQ-II score suggests that this compensatory effect may become stronger as the FOG progresses.

In addition, we found that the impaired performances in FOG (first step range of motion, stride length, and turn steps) were all correlated with the ALFF value changes in the bilateral sensorimotor regions and globus pallidus. According to the “interference model” mentioned above, abnormally increased inhibitory outputs originate from the internal pallidum lead to an excessive inhibition of the thalamus and brainstem; consequently, a freezing episode might be triggered^19^. According to the classic basal ganglia model^43^, basal ganglia dysfunction leads to the excessive inhibition in the sensorimotor areas, which in turn results in some motor deficits in PD (e.g., akinesia).

We also found that the first step range of motion was negatively correlated with ALFF value in the left middle temporal gyrus. Functions of the middle temporal gyrus might be involved in conflict resolution of multiple inputs from vestibular and other sensory afferents during gait initiation^44^. Our findings indicate that abnormal activity in the temporal cortex is associated with the impaired gait initiation in PD patients with FOG. The stride length during straight walking was associated with abnormal activity within the temporal and occipital cortices. These areas contribute to the integration of visual sensory information, which is damaged in FOG^45^. Using FDG-PET, Lyoo *et al*. reported that metabolism within these regions was decreased in PD patients with FOG, and such hypometabolism limited the efficiency of STN-DBS on FOG^46^. Additionally, we found that turn steps were associated with activity in the left precuneus. One recent resting-state fMRI study also demonstrated that hyper-connectivity involving the precuneus was correlated with a higher step number during dual-task turning^47^. Both of these studies suggested that abnormal activity in the precuneus might play a role in turning in patients with FOG.

It has been generally suggested that FOG is significantly correlated with cognitive decline, particularly executive dysfunction^37^. Unfortunately, we did not find any significant difference in the total score or the Executive Function sub-score of MoCA among the three groups. This discrepancy might be attributed to the fact that we only assessed MoCA to evaluate the cognitive function, rather than using other assessments which are specific to test the executive function. Despite this limitation, we found that the spontaneous activity in the right Crus I of cerebellum had negative correlation, while the spontaneous activity in the left SFG and left putamen had positive correlations with the Executive Function sub-score. Previous functional imaging studies showed that the underactivity and pathological disorganization within the frontostriatal networks are involved in the executive dysfunction in PD patients^48,49^. Lewis *et al*.^48^, using event-related fMRI, found that the executively impaired PD patients exhibit significantly less activation in the dorsolateral and ventrolateral prefrontal cortex as well as putamen compared to patients with no such deficit. Consistent with their study, our finding of positive correlation with ALFF in the SFG and putamen demonstrated that the spontaneous activity within these regions decreased as the executive dysfunction progresses. Additionally, the involvement of cerebellum in the executive functions has been well established^50–52^, and the interconnection between the cerebellum and the prefrontal cortex plays an important role in the pathogenesis of executive function impairment^50,53^. In the present study, the negative correlation between the ALFF in the Crus I of cerebellum and Executive Function sub-score might be a reflection of the cerebellar compensatory effect to maintain the executive function in patients with FOG. Further studies are needed to investigate the executive dysfunction in PD patients with FOG.

Our study has some limitations that should be considered. First, our patients with FOG had greater disease severity, longer duration, and greater LEDD compared to patients without FOG. As we asked the patients to withdraw the medications at least 12 h before fMRI scanning, the confounding from mismatched levodopa dosage could be reduced. Moreover, to minimize the above potential confounds, we included MDS-UPDRS III, H-Y stage, disease duration, and LEDD as covariates when comparing the ALFF values between patients with FOG and without FOG. Second, the gait analyses performed in the present study are an indirect reflection of FOG. We did not measure other objective measures except for the NFOGQ (for instance, the percentage of time spent freezing during the Stand and Walk trials). Third, we did not evaluate assessments that are specific to the executive function, as discussed above. Future studies with these measurements are warranted to improve our understanding on FOG.

In conclusion, the present study demonstrates that there are spontaneous brain activity changes in the resting state in PD patients with FOG. Our findings suggest that FOG in PD is associated with impairment within frontal and parietal regions, along with increased basal ganglia inhibitory output. Also, we proposed that cerebellar dysfunction might be involved in the pathophysiology of FOG in PD patients. Future investigation of longitudinal changes in brain activity during the progression of FOG may provide more information.

## Methods

### Participants

We studied 62 PD patients (31 with FOG and 31 without FOG) recruited from the Movement Disorders Clinic of the Xuanwu Hospital of Capital Medical University. Exclusion criteria were: history of deep brain stimulation surgery; marked rest tremor; presence of contraindications for MRI; MMSE score ≤24; comorbidities of neurological disease other than PD. A group of 32 healthy sex- and age-matched volunteers served as controls. The study was approved by the local Medical Ethics Committee and written informed consent was obtained from all participants prior to the experiment. The experiments were performed according to the Declaration of Helsinki and were approved by the Institutional Review Board of Xuanwu Hospital.

Patients were classified as “with FOG” and “without FOG” based on the positive response to Part I of the NFOGQ^54^, a dichotomous question that asks whether FOG episodes were experienced during the past month or not. Recognition of FOG episodes was illustrated by the presentation of a short video (70 s) demonstrating typical freezing phenotypes. NFOGQ has previously been shown to be a reliable tool to detect FOG^55^. In 27 of the 31 (87%) self-reported freezers, FOG was confirmed during clinical testing or spontaneous behavior. None of the patients classified as non-freezers demonstrated FOG episodes during the physical examination.

### Clinical assessments

Clinical assessments of patients were conducted in their practical off state, that is, at least after a 12-hour withdrawal of anti-Parkinson medication. The MDS-UPDRS III and H-Y stage assessed motor disability and disease severity. MoCA assessed general cognitive function^56^, and HAMD measured affective symptoms. Moreover, the executive domain of MoCA (trail making, phonemic fluency, and verbal abstraction; 4 points) was evaluated to assess executive function^56^. Parts II and III of the NFOGQ were used to evaluate FOG severity and disability, respectively.

### Quantitative gait analysis

All participants underwent quantitative assessments of gait using Opal inertial sensors, Mobility Lab, the clinical user interface and automated algorithms by Ambulatory Parkinson’s Disease Monitoring (APDM Inc. http://apdm.com). Subjects wore six Opal sensors composed of 3D accelerometers, 3D gyroscopes, and 3D magnetometers. The sensors were positioned with Velcro belts on the posterior trunk, on the anterior shank of each leg, on each wrist, and on the sternum. Data were acquired and automatically analyzed with MobilityLab^57,58^. All participants performed four trials of the Instrumented Stand and Walk test, designed to assess several domains of gait initiation, straight walking and turning^59^. The Instrumented Stand and Walk test consisted of standing quietly for 30 seconds, followed by initiating gait with the most involved leg or dominant leg, walk 7 meters, turn 180 degrees after crossing a line on the ground, and return to the initial position. During quiet standing, subjects were asked to keep their arms at their sides and look straight ahead. A template was used to achieve consistent foot placement with 10 cm between heels and a 30-degree outward rotation of the feet^58^. We measured gait initiation (anticipatory postural adjustments duration and latency, first step range of motion and first step latency), straight walking (stride length, stride velocity, cadence, double support time%, swing time %, stance time % and arm swing amplitude) and turning (turn duration, turn steps and peak velocity) in each subject.

### Functional MRI acquisition

Imaging was carried out in a SIEMENS Trio 3 T scanner. Participants were instructed to keep their head still and eyes closed during scanning. Earplugs and a head coil with foam pads were used to minimize machine noise and head motion. fMRI scans were acquired following a 12-hour period of medication withdrawal in all patients. For each participant, we acquired high-resolution T1- and T2-weighted anatomical images, and a radiologist viewed the images to exclude participants with space-occupying lesions and cerebrovascular diseases. BOLD images were obtained using the following SE-EPI sequence: repetition time = 2000 ms, echo time = 30 ms, voxel size = 3.0 × 3.0 × 3.0 mm^3^, slice thickness/gap = 4.0/0 mm, axial slices = 33 layers, flip angle = 90°, FOV = 256 mm × 256 mm, matrix size = 64 × 64, and scanning time = 8 min.

### Data preprocessing

Data were preprocessed and analyzed using DPABI version 2.2 (http://rfmri.org/dpabi) and SPM12 software package (http://www.fil.ion.ucl.ac.uk/spm). The first 10 time points were discarded to allow the magnetization to approach a dynamic equilibrium and to allow participants to get used to the scanning noise. The remaining images were corrected for slice timing with the middle slice as a reference, realigned to remove excessive head motion. EPI data were then normalized into a standard brain space template (the Montreal Neurological Institute template), and resampled to 3.0 × 3.0 × 3.0 mm isotropic voxels. Linear trends were removed and images were smoothed with a 6-mm Gaussian kernel to increase the signal-to-noise ratio. We further reduced potential confounds of head motion with Friston-24 correction using Friston24-parameter model (6 head motion parameters, 6 head motion parameters one time point before and the 12 corresponding squared items^60^). To reduce possible effects of physiological artifacts, the nuisance covariates of cerebrospinal fluid signal and white matter signal were finally regressed-out. For each participate, the instantaneous head motion was calculated along with frame-wise displacement (FD) as defined by Jenkinson *et al*.^61^, which was preferable for its consideration of voxel-wise differences in its derivation^62^. Subjects were excluded from analysis if their head motion (mean FD Jenkinson) was greater than mean + 2 * SD (threshold 0.25 mm)^63^. FD correction led to exclusion of 6 participants (patients with FOG: n = 2; patients without FOG: n = 3; healthy controls: n = 1).

### ALFF calculation

ALFF calculation was performed using RS-fMRI Data Processing Toolkit REST version 1.8 (REST, http://rest.restfmri.net)^64^. Each time series was transformed to the frequency domain through fast Fourier transform. The square root of the power spectrum was computed and the averaged square root obtained across 0.01–0.08 Hz was taken as the ALFF measurement. The resultant ALFF of each voxel value was then further divided by the global mean value for standardization.

### Statistical analysis

#### Clinical characteristics

Demographic data were presented as mean ± SD for continuous variables. Independent two samples t-test and one-way analysis of covariance (ANOVA) were performed for the comparison of continuous variables, and the chi-squared test was used to compare categorical variables. *Post-hoc* analyses were then used to assess group differences in performance on MMSE, MoCA, and HAMD, as well as all the metrics of gait derived from the Opal sensors. The threshold for the level of significance was set at α = 0.05.

#### Functional imaging analysis

One-way ANOVA was performed to identify differences of regional ALFF values among patients with FOG, patients without FOG and healthy controls. Brain areas showing significant differences (voxel-level p < 0.001, cluster size >945 mm^3^/35 voxels, corresponding to a corrected p < 0.05 as determined by AlphaSim correction) in the ANOVA analysis were then extracted as a mask. *Post-hoc* analyses within this mask were used to explore pair-wise differences among the three groups. To account for the differences in motor severity, disease duration, H-Y stage, MDS-UPDRS III score and LEDD, these features were applied as covariate variables in the comparison between patients with and without FOG. The same AlphaSim correction was also used in the *post-hoc* analyses.

To explore the relationship between brain activity and the severity of FOG, Pearson’s correlation was computed between the ALFF values and several FOG measures, including the subjective measure (NFOGQ-II) as well as the objective measures/gait parameters which were more pronounced impaired or specific in patients with FOG. As shown in the Results of Gait Performances/Table 2, the first step range of motion, stride length, stride velocity and turn peak velocity were more pronounced impaired in PD patients with FOG, while turn duration, turn steps and arm swing amplitude were relatively specific to FOG. We then chose three of the above parameters from the three different gait domains to be performed in the correlation analyses, that is, “first step range of motion” from step initiation, “stride length” from straight walking, and “turn steps” from turning. In addition, although no significant difference was found among the three groups, we also performed the correlation analysis between ALFF values and Executive Function sub-score due to the cognitive nature of FOG^36–38^.

### Data availability

The datasets generated during and/or analyzed during the current study are available from the corresponding authors on reasonable request.
