# Author Correction: Altered resting-state brain activity in Parkinson’s disease patients with freezing of gait

**DOI:** 10.1038/s41598-018-23233-5

**Published:** 2018-03-14

**Authors:** Tao-Mian Mi, Shan-Shan Mei, Pei-Peng Liang, Lin-Lin Gao, Kun-Cheng Li, Tao Wu, Piu Chan

**Affiliations:** 1 Department of Neurology, Neurobiology and Geriatrics, Xuanwu Hospital of Capital Medical University, Beijing Institute of Brain Disorders, Beijing, 100053 China; 2 Department of Radiology, Xuanwu Hospital of Capital Medical University, Beijing, 100053 China; 3 Beijing Key Laboratory of MRI and Brain Informatics, Beijing, 100053 China; 4 Clinical Center for Parkinson’s Disease, Capital Medical University, Beijing, 100053 China; 5 Key Laboratory for Neurodegenerative Disease of the Ministry of Education, Beijing Key Laboratory for Parkinson’s Disease, Beijing, 100053 China; 6 National Clinical Research Center for Geriatric Disorders, Beijing, 100053 China

Correction to: *Scientific Reports* 10.1038/s41598-017-16922-0, published online 01 December 2017

This Article contains errors in the Author Contribution Statement.

“Chan P. and Wu T. designed the experiments and revised the manuscript; Mi T., Mei S. and Gao L. carried out data collection; Mi T. carried out data analysis and drafted the manuscript; Liang P. and Li K. carried out functional fMRI scan”.

should read:

“Chan P., Wu T. and Li K. designed the experiments and revised the manuscript; Mi T., Mei S. and Gao L. carried out data collection; Mi T. carried out data analysis and drafted the manuscript; Liang P. carried out functional MRI scan and data analysis”.

